# Comparative Immunogenicity of Evolved V1V2-Deleted HIV-1 Envelope Glycoprotein Trimers

**DOI:** 10.1371/journal.pone.0067484

**Published:** 2013-06-26

**Authors:** Ilja Bontjer, Mark Melchers, Tommy Tong, Thijs van Montfort, Dirk Eggink, David Montefiori, William C. Olson, John P. Moore, James M. Binley, Ben Berkhout, Rogier W. Sanders

**Affiliations:** 1 Department of Medical Microbiology, Laboratory of Experimental Virology, Center for Infection and Immunity Amsterdam (CINIMA), Academic Medical Center, Amsterdam, The Netherlands; 2 Torrey Pines Institute for Molecular Studies, San Diego, California, United States of America; 3 Department of Surgery, Duke University Medical Center, Durham, North Carolina, United States of America; 4 Progenics Pharmaceuticals, Tarrytown, New York, United States of America; 5 Department of Microbiology and Immunology, Weill Medical College of Cornell University, New York, New York, United States of America; Shanghai Medical College, Fudan University, China

## Abstract

Despite almost 30 years of research, no effective vaccine has yet been developed against HIV-1. Probably such a vaccine would need to induce both an effective T cell and antibody response. Any vaccine component focused on inducing humoral immunity requires the HIV-1 envelope (Env) glycoprotein complex as it is the only viral protein exposed on the virion surface. HIV-1 has evolved several mechanisms to evade broadly reactive neutralizing antibodies. One such a mechanism involves variable loop domains, which are highly flexible structures that shield the underlying conserved epitopes. We hypothesized that removal of such loops would increase the exposure and immunogenicity of these conserved regions. Env variable loop deletion however often leads to protein misfolding and aggregation because hydrophobic patches becoming solvent accessible. We have therefore previously used virus evolution to acquire functional Env proteins lacking the V1V2 loop. We then expressed them in soluble (uncleaved) gp140 forms. Three mutants were found to perform optimally in terms of protein expression, stability, trimerization and folding. In this study, we characterized the immune responses to these antigens in rabbits. The V1V2 deletion mutant ΔV1V2.9.VK induced a prominent response directed to epitopes that are not fully available on the other Env proteins tested but that effectively bound and neutralized the ΔV1V2 Env virus. This Env variant also induced more efficient neutralization of the tier 1 virus SF162. The immune refocusing effect was lost after booster immunization with a full-length gp140 protein with intact V1V2 loops. Collectively, this result suggests that deletion of variable domains could alter the specificity of the humoral immune response, but did not result in broad neutralization of neutralization-resistant virus isolates.

## Introduction

The need for an effective HIV-1 vaccine is undisputed, but the challenges in the development of such a vaccine are formidable. Recently, one vaccine candidate showed some degree of protection in the RV144 phase III trial [1], although the mode of protection is not yet entirely clear and it is questionable whether the use of a vaccine with only 31% efficacy would have a significant effect on the epidemic [2]. Thus, there is a need for improved vaccines.

Traditional antiviral vaccines typically consist of live-attenuated or inactivated virus as these are usually effective in achieving protection against subsequent infection. Although live-attenuated SIV/HIV was shown to induce protection against infection, it is not considered safe for public use because of the risk of reversion of the vaccine strain to a pathogenic phenotype [3], [4], [5], [6]. Inactivated SIV/HIV is safe, but was found to be ineffective in raising a sufficiently neutralizing antibody response [7]. Effective subunit protein vaccines have been developed for hepatitis B virus (HBV) and human papillomavirus (HPV) [8], [9], but HIV-1 protein subunit vaccines have not been effective so far [10], [11].

A vaccine aimed at generating an humoral response against HIV-1 would have to include at least some component of the envelope glycoprotein complex (Env), because it is the only viral protein accessible for antibodies on the intact virus particle surface and therefore the only component able to induce neutralizing antibodies (NAbs). The functional HIV-1 Env complex is a heterotrimer consisting of 6 subunits; three gp120 and three gp41 molecules. Collectively, the gp120 and gp41 molecules mediate entry of HIV-1 into CD4^+^ T cells. Since the surface subunit gp120 is a relatively large component of the Env complex compared to the transmembrane subunit gp41 and the complex is not stable as a whole, at least in soluble form, initially Env subunit vaccines were tested containing only the gp120 Env component. However, these did not induce protective immune responses including neutralizing antibodies [12], [13], emphasizing the need for more sophisticated Env immunogens.

Env has evolved several defense mechanisms to limit the induction of neutralizing antibodies. One such mechanism is the abundant exposure of immunodominant “decoy” epitopes on non-functional forms of Env that induce non-neutralizing antibodies that do not recognize the functional Env trimer [14], [15], [16], [17]. Non-functional Env forms derive from various sources, including dissociation of the functional Env complex, resulting in exposed gp41 and gp120. As a consequence, the antibody response is dominated by non-neutralizing specificities, both in naturally infected individuals as well as individuals vaccinated with gp120 subunit immunogens.

Another defense mechanism developed by HIV-1 is the presence of several highly variable protein loops (V1–V5) that cover the more conserved protein components of gp120 from recognition by antibodies. These variable loops are generally immunodominant and although their recognition by antibodies can lead to neutralization, in most cases, the virus can easily escape from these effects by acquiring mutations that do not jeopardize Env function. However, the more conserved parts of the variable domains can be targeted by broadly neutralizing antibodies, as demonstrated for the recently identified broadly neutralizing PG9 and PG16 antibodies that target the V1V2 domain [18], [19], [20]. Furthermore, it was shown recently that V2-specific antibodies correlated with protection in the RV144 trial and exerted selection pressure on the transmitted virus [21], [22].

A third protection mechanism is the use of a “glycan shield” that decorates the outside of the functional Env trimer complex [23], [24]. The Env amino acid sequence harbors 24–37 consensus sites for N-linked glycan attachment depending on the virus isolate, most of which are used [25], [26]. Approximately half of gp120’s molecular weight consists of glycans, and gp41 is also glycosylated. Because the *N*-glycans on Env are synthesized by the host cell protein glycosylation machinery, these are usually not considered as foreign by the immune system. By hiding its critical epitopes underneath *N*-glycans, HIV-1 effectively prevents the induction of a neutralizing antibody response to these protein domains. The recent discovery of many broadly neutralizing antibodies with glycan-dependent epitopes has revised the above view and showed that the human humoral immune system can actually pierce HIV-1′s glycan shield [18], [19], [20], [25], [26], [27], [28]. Furthermore, some studies suggest that the oligomannose residues of the glycan shield play a role in interfering with dendritic cell (DC) function [29], [30], [31], [32], which may contribute to the inefficient initiation of an antibody response against gp120 and may explain why antibody levels wane quickly. As many *N*-glycans are anchored to the variable loops, these protection mechanisms of glycans and variable loops are interlinked.

To solve the instability of the Env complex and to prevent the exposure of non-neutralizing decoy epitopes on nonfunctional Env forms, we and others have engineered recombinant versions of the native, trimeric complex. Our approach has been to stabilize the gp120-gp41 (SOS gp140; [33] and gp41-gp41 (SOSIP gp140; [34]) interactions, while ensuring precursor cleavage [35]. Stabilized, soluble Env trimers still contain the variable loop and glycan shield defenses, which could be one reason why they are only slightly better at inducing neutralizing antibodies than monomeric gp120.

Although conserved elements of the V1V2 domain can harbor potentially protective epitopes [18], [19], [20], [21], [22], deletion of the V1V2 loops can also be a means of improving the exposure of other neutralizing epitopes and enhancing the immunogenicity of gp120 [36], [37], [38], [39]. We have also attempted to develop Env variants with a reduced number of variable loops, but deletion of variable loops proved problematic in the context of soluble Env trimers because of misfolding, aggregation and other problems associated with the aberrant exposure of hydrophobic domains ([40], [41], [42], [43], [44] and unpublished results). Virus evolution experiments with ΔV1V2 gp160 in the context of live virus proved successful at largely overcoming these obstacles, leading to ΔV1V2 variants with greatly improved function due to the selection of compensatory mutations near the ΔV1V2 stump. Three mutants performed most effectively in terms of stability and expression levels; ΔV1V2.2, ΔV1V2.4.DNGSEK and ΔV1V2.9.VK [40], [41].

The efficient expression of soluble ΔV1V2 Env gp140 trimers allowed us to test their immunogenicity in rabbits. Here we characterize the immune response to these three V1V2 deletion mutants compared to wild-type Env. All variants induced antibodies against gp120 and trimeric Env. Env ΔV1V2.9.VK induced the highest antibody titers to neo-epitopes (specific for the immunogen only), but also induced stronger neutralization of the SF162 strain than wild-type Env at early time points. This suggests that refocusing of the immune response to other epitopes on Env is feasible by means of deletion of the V1V2 region, although it did not result in neutralization of the more resistant virus isolates.

## Results

### Design and Construction of Evolved ΔV1V2 Env Trimers

**Figure 1:**
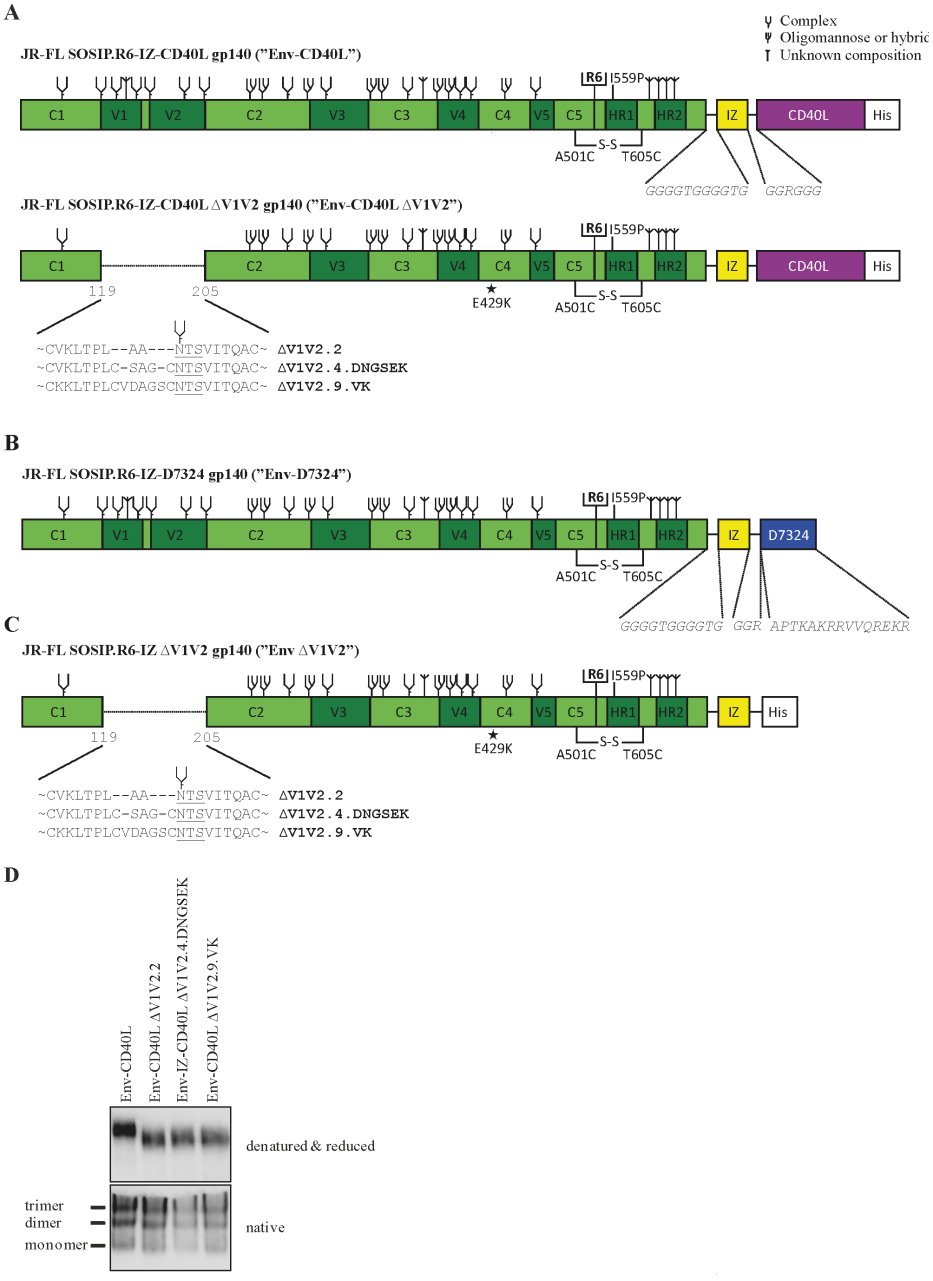
Env ΔV1V2 mutant design and expression. (**A**) Linear representation of Env and the mutants Env ΔV1V2.2, ΔV1V2.4.DNGSEK and ΔV1V2.9.VK. The clade B JR-FL gp140 (amino acids 31–681) contains several modifications that have been indicated in the schematic (see materials and methods and results sections for more details). (**B**) Schematic of the D7324-tagged JR-FL used in ELISAs. (**C**) Schematic of the His-tagged ΔV1V2.2 JR-FL gp140 used in ELISAs. (**D**) Reducing SDS-PAGE (upper panel) and Blue Native-PAGE (lower panel) analysis of full length Env, Env ΔV1V2.2, ΔV1V2.4.DNGSEK and ΔV1V2.9.VK secreted from transiently transfected HEK 293T cells.

In order to refocus the antibody response from variable domains towards more conserved epitopes of the HIV-1 Env protein, variable domains were removed. Several studies have investigated removal of the V3 or V4 region, but this resulted in a total loss of Env function and virus infectivity [40], [45], [46], [47], [48]. Deletion of the V1V2 variable domain also results in a loss of Env function, but we previously selected improved virus variants with restored Env function by compensatory amino acid substitutions. Three ΔV1V2 trimer constructs, ΔV1V2.2, ΔV1V2.4.DNGSEK and ΔV1V2.9.VK behaved best in terms of Env function, trimer expression and protein stability [40], [41](Fig. 1). These are shown in Fig. 1. The ΔV1V2.2 construct was originally designed such that the disulfide bond between residues C126 and C196 was replaced by two alanines to create a continuous protein backbone between these two residues. Two variants, ΔV1V2.4.DNGSEK and ΔV1V2.9.VK, retained cysteines C126 and C196 and a few amino acids in between, but they contain compensatory changes that neutralize a hydrophobic patch at the V1V2 stem that becomes exposed upon V1V2 deletion [40], [41]. The G127S substitution in variant ΔV1V2.4.DNGSEK and the G128D and V120K substitutions in variant ΔV1V2.9.VK reduce the hydrophobic patch by substituting a hydrophobic amino acid for a hydrophilic one. The D197N substitution in ΔV1V2.4.DNGSEK generates a glycosylation site and the attached *N*-glycan probably covers the hydrophobic patch.

These three variants were introduced into a stabilized gp140 construct based on the CCR5-using clade B isolate JR-FL. The various modifications to stabilize gp140 trimers have all been described in detail [33], [34], [35], [49], [50], [51](Fig. 1A–C). To target the ΔV1V2 gp140 trimers to dendritic cells and B cells to enhance immune responses [51], we added the sequences of rabbit CD40 ligand (CD40L) to the C-terminus of gp140. However, we found in concurrent studies that the antibody responses were more efficiently enhanced by fusion of Env to the B cell activating molecule A PRoliferation-Inducing Ligand (APRIL) [50].

To investigate the expression level and stability of the ΔV1V2 fusion proteins, the proteins were transiently expressed in HEK 293T cells and analyzed by SDS-PAGE and Blue Native PAGE (BN-PAGE). All constructs were secreted efficiently from HEK 293T cells, although the expression of the ΔV1V2 variants seemed slightly reduced compared to the control construct containing a full-length V1V2 domain (Fig. 1D, upper panel). The ΔV1V2 constructs migrated faster through the gel, consistent with the reduction in molecular weight of approximately 20 kDa (140 kDa vs 120 kDa) (Fig. 1D, upper panel). Note that the fusion constructs are not cleaved since adding protein domains to the C-terminus of JR-FL SOSIP.R6 gp140 trimers renders them uncleavable [51]. BN-PAGE analysis showed that all fusion proteins were predominantly trimeric although considerable amounts of dimer and trace amounts of monomer were also visible (Fig. 1D, lower panel).

### Evolved ΔV1V2 Trimers Induce Env-specific Antibody Responses

**Figure 2:**
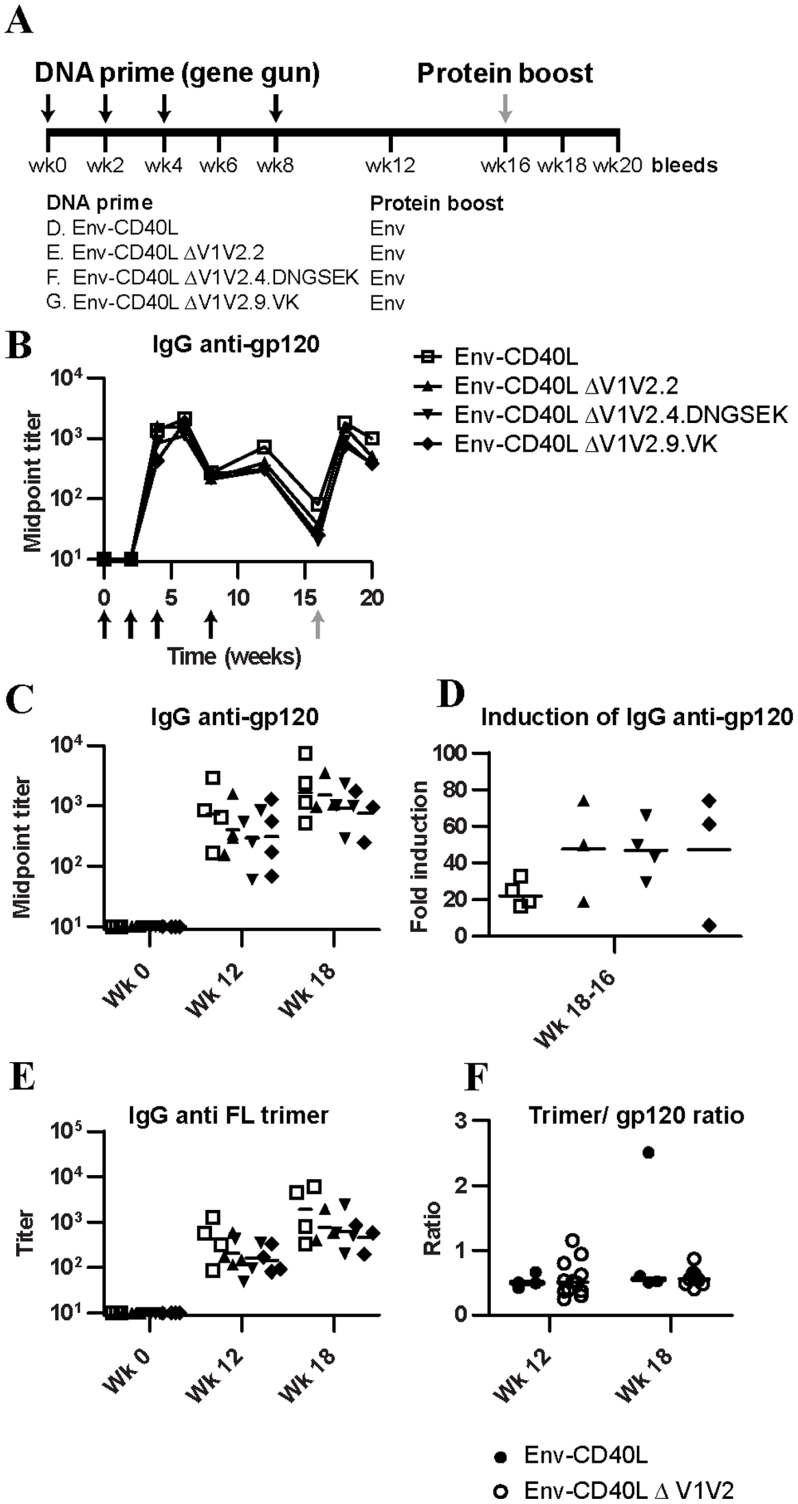
Study design and antibody titers. (**A**) Immunization scheme. Rabbits were primed with DNA constructs expressing the full length or ΔV1V2 variants. All groups were boosted with full length, cleaved JR-FL SOSIP.R6 gp140 protein [34], [54] in Quil A adjuvant. (**B**) Midpoint IgG anti-gp120 titers in the rabbit sera over the course of the experiment as determined by ELISA. (**C**) Midpoint IgG gp120-binding titers at week 0, 12 and 18. (**D**) Fold-induction of gp120 binding titers upon protein boosting (i.e. week 18 titers vs week 16 titers). (**E**) Midpoint IgG binding titers againt full-length trimeric gp140 at week 0, 12 and 18. (**F**) Ratio of trimer/gp120 binding titers.

To test whether V1V2 deletion improves the induction of neutralizing antibody responses, we compared the antibody responses induced by the different constructs in a rabbit immunization experiment. We used rabbits because, in contrast to mice, their antibodies have longer CDR H3 domains that are commonly found in human broadly neutralizing antibodies against HIV-1 [52], [53]. Four groups of four rabbits were immunized with plasmid DNA encoding one of the three ΔV1V2 constructs or with the V1V2-containing parental control. Note that this control group is the same as group D described in reference [50]. These studies were conducted simultaneously to reduce the number of control animals. Immunizations were performed by gene gun at wk 0, 2, 4 and 8 (Fig. 2A). At week 16, all rabbits were boosted with the same protein immunogen; cleaved JR-FL SOSIP.R6 gp140 [34], [54]) in Quil A adjuvant. The rationale for this choice of boosting protein was two-sided. First, we hypothesized that boosting with a full-length Env might boost antibody specificities generated by ΔV1V2 Env that recognize the full-length trimer. Second, we envisioned that boosting with a pure cleaved protein might selectively boost responses that recognize the cleaved Env protein.

Immune sera were tested for their capacity to bind parental V1V2-containing gp120 in ELISA. The anti-gp120 binding IgG titers induced by the three ΔV1V2 constructs were slightly lower than those induced by full-length Env (Fig. 2B–C), both before and after the full length protein boost. Although this difference was not significant, it may reflect the slightly reduced expression levels (Fig. 1D, upper panel). Alternatively, this could be explained by the lack of antibody responses against the V1V2 domain. After boost immunization with cleaved JR-FL SOSIP.R6 gp140, titers rose to comparable levels in all groups (Fig. 2B–C), but the level of induction was not significantly different (Fig. 2D).

Next, we tested for the presence of trimeric Env-binding antibodies, using a full-length stabilized trimeric JR-FL SOSIP.R6 gp140 with a C-terminal D7324 epitope that allows efficient capture in ELISA (“Env-D7324”; Fig. 1B) [49], [50]. All four constructs induced anti-trimer IgG responses (Fig. 2E). Again the responses induced by the ΔV1V2 Env variants were slightly lower than those triggered by full-length Env, but the difference was not significant (Fig. 2E,F). We also determined the total IgG levels in the sera, which were found to be very similar (data not shown).

### Evolved ΔV1V2 Trimers do not Induce Broadly Neutralizing Antibody Responses

**Figure 3:**
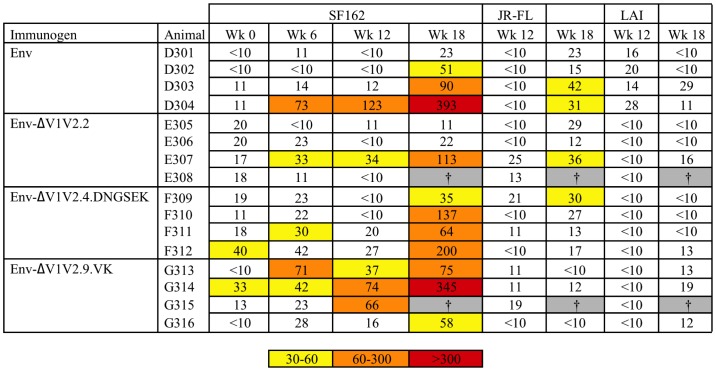
50% neutralization titers against SF162, JR-FL and LAI. Data are from the Academic Medical Center. Midpoint neutralizing titers against SF162 at week 0, 6, 12 and 18 and midpoint neutralizing titers against LAI and JR-FL virus at week 12 and 18. The titer data are colored according to the following color scale: yellow, 50% neutralization titers between 30 and 60; orange, between 60 and 300; red, >300. † Animals died of unrelated causes between week 12 and week 18.

We next studied the sera for virus-neutralizing capacity against various tier 1 and tier 2 HIV-1 strains. We first tested the ability of the rabbit sera to neutralize the SF162 strain in a single cycle infection assay based on TZM-bl reporter cells (Fig. 3). SF162 is a highly neutralization sensitive strain that is classified as tier 1 [55], [56]. For instance, SF162 is relatively sensitive to V3-directed antibodies. Because of this ultra-sensitivity, SF162 neutralization can be a useful tool to detect early responses and subtle differences. As expected, neutralization by the pre-bleed control samples was low and the neutralization titers gradually increased at weeks 6 and 12. The ΔV1V2.9.VK-induced sera neutralized SF162 more efficiently than the three other immunization groups at weeks 6 and 12. Most sera were able to efficiently neutralize SF162 at week 18 after the SOSIP.R6 gp140 boost, and no differences were apparent anymore among the sera.

Next, we tested the more neutralization-resistant tier 2 JR-FL strain [55], which is homologous to the immunogen (Fig. 3). JR-FL neutralization titers were low at week 12, but were somewhat increased by week 18. Env ΔV1V2.9.VK did not induce significant neutralization of JR-FL, even at week 18. Env ΔV1V2.2 and Env ΔV1V2.4.DNGSEK induced titers >25 in 2/3 and 2/4 rabbits per group, respectively. Neutralization of the CXCR4-using LAI strain was also analyzed. LAI is the strain in which the original ΔV1V2 mutants were created (see below) [40], [41]. Neutralization of LAI was only sporadically observed (Fig. 3). At both week 12 and 18 there was one serum that was able to neutralize at titers >25, but these were from different animals, both of which belonged to the full-length Env immunization group. The mildly better neutralization by the full-length Env immunized animals was significant (p<0.05) for the week 12 sera.

**Figure 4:**
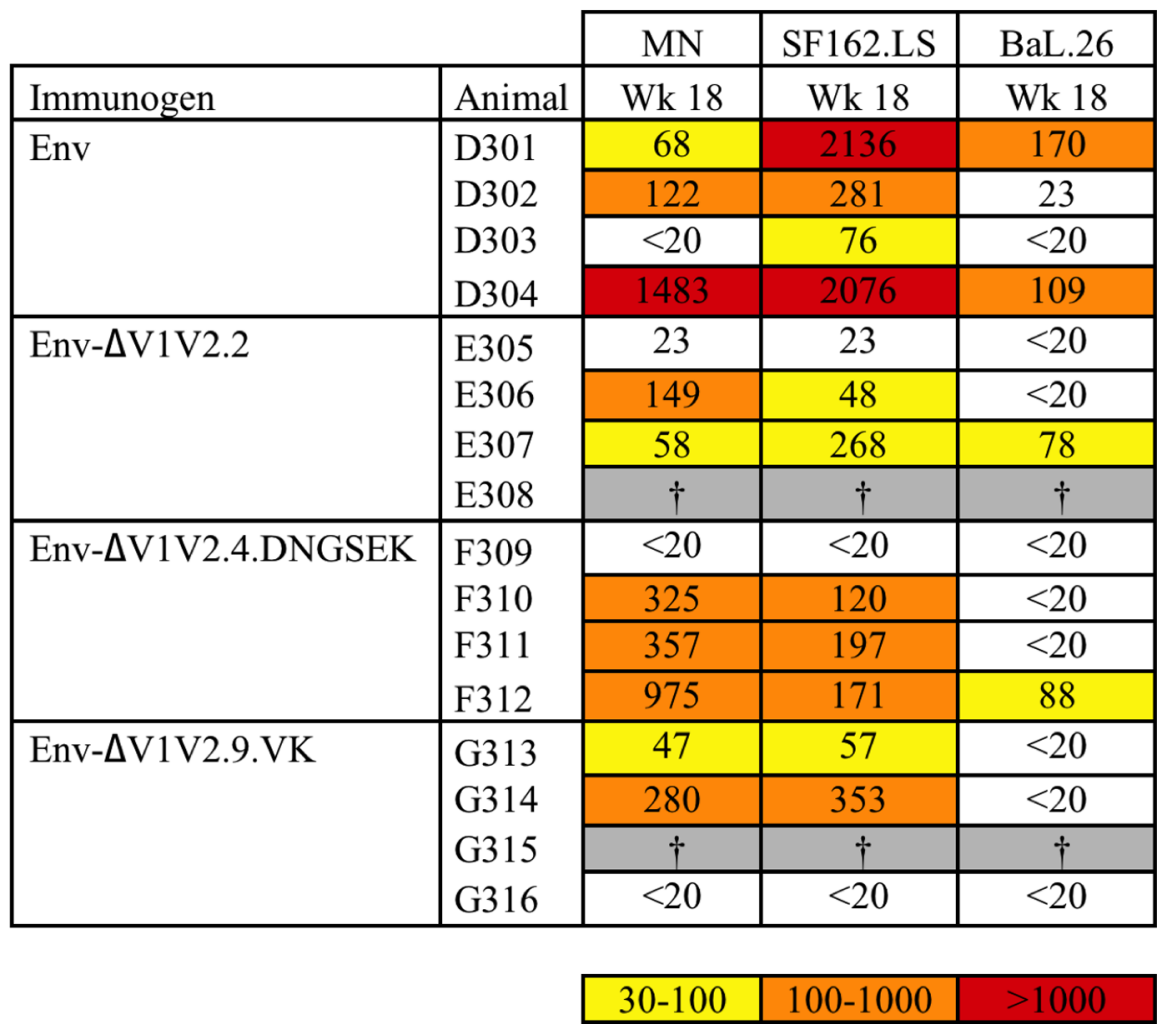
50% neutralization titers against tier 1 viruses. Data are from the Central Immunology Laboratory. Midpoint neutralizing titers of MN, SF162.LS and Bal.26 were measured for week 18 sera. The titers are colored according to the following color scale: yellow, 50% neutralization titers between 30 and 100; orange, titers between 100 and 1,000; red, >1,000. ^†^Animals died of unrelated causes between week 12 and week 18.

These results were largely corroborated by independent analysis of the week 18 serum samples at the Duke Central Immunology Laboratory for AIDS Vaccine Research and Development (Fig. 4). Although the neutralization titers obtained were generally higher than with our in-house assay, which may be caused by different *env* clones used, the trends observed for the SF162.LS and BaL.26 virus strains were similar, with Env inducing most efficient neutralization of these two strains at week 18. Three out of four sera from rabbits treated with full-length Env neutralized SF162.LS at titers >250, whereas one out of four Env ΔV1V2.2 and ΔV1V2.9.VK sera were able to do so. None of three ΔV1V2.4.DNGSEK sera neutralized SF162.LS at titers >250. The control Env immunization group was also able to neutralize the BaL.26 strain most efficiently with 2/4 sera having titers >100. Sera generated against ΔV1V2.2, ΔV1V2.4.DNGSEK and ΔV1V2.9.VK gp140 showed reduced BaL.26 neutralization activity, with 1/3, 1/4 and 0/3 sera, respectively, having titers >50. The patterns were somewhat different for the tier 1 MN strain, which was neutralized most efficiently by ΔV1V2.4.DNGSEK-induced sera with 3/4 sera having titers >250. The other immunogens performed less well, showing neutralization with 1/4, 1/3 and 0/3 sera for Env, Env ΔV1V2.9.VK and Env ΔV1V2.2, respectively. We did not observe neutralization of the tier 2 viruses JR-FL, 6535.3, QH0692.42, PVO.4 and RHPA4259.7 (data not shown).

Other serum factors can influence the apparent neutralization capacity, such as IFNγ or factors influencing cell viability. In order to rule out such effects, sera samples from rabbits E307 (Env ΔV1V2.2), F312 (Env ΔV1V2.4.DNGSEK) and G314 (Env ΔV1V2.9.VK) were IgG-depleted using protein G-coupled agarose beads. This depleted serum containing less than 5% of original IgG level (data not shown) was tested for standard SF162 neutralization and compared with the (semi-purified) IgG eluted from the beads (75% recovery) (data not shown) and the original, untreated serum. The depleted sera were unable to neutralize SF162 to a significant level (data not shown), whereas the purified IgG and unfractionated sera were able to do so (data not shown).

**Figure 5:**
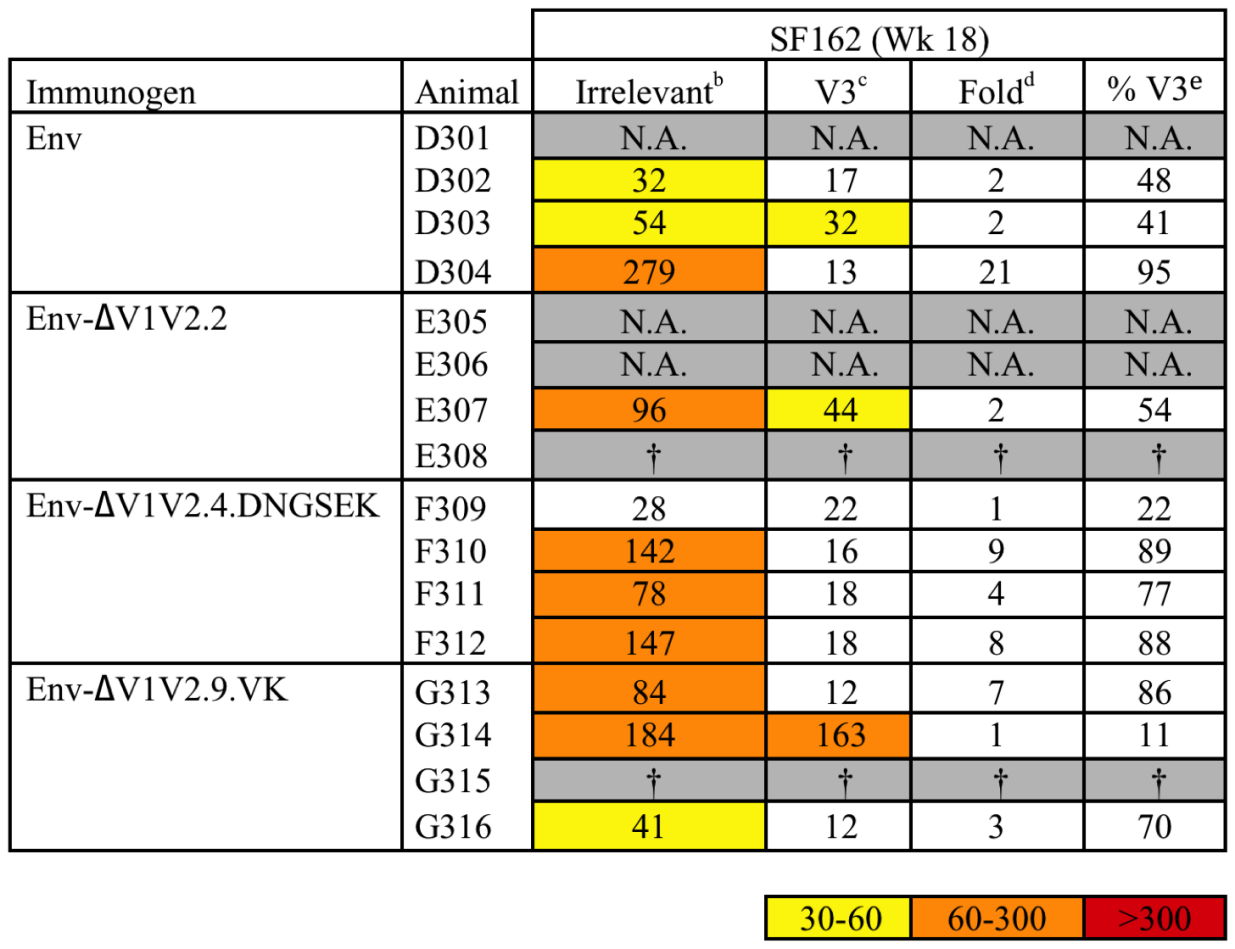
V3-peptide depletion of SF162 neutralization. Midpoint neutralizing titers against SF162 at week 18 in the absence and presence of V3-peptides. Experimental conditions are similar to those in Fig. 3. The titer data are colored according to the following color scale: yellow, 50% neutralization titers between 30 and 60; orange, between 60 and 300; red, >300. ^a^ Values represent 50% neutralization titers in the presence of irrelevant peptide. ^b^ Values represent 50% neutralization titers in the presence of V3 peptides. ^c^ Values represent fold decreases in 50% neutralization titers caused by V3 peptides. ^d^ Values represent percentages of V3-specific neutralization. †Animals died of unrelated causes between week 12 and week 18. N.A., not analyzed; sera from these rabbits did not neutralize SF162 potently in earlier experiments (see Fig. 3).

Tier 1 virus neutralization is often dominated by anti-V3 antibodies [55]. It is therefore conceivable that V1V2 deletion may skew additional responses to the V3. To test for this possibility, we performed SF162 neutralization experiments in the presence of interfering V3 peptides or an unrelated control peptide (Fig. 5). The 50% SF162 neutralization titers in the presence of the control peptide were comparable to those obtained in the absence of any peptide (compare Figs. 3 and 5). In contrast, the 50% neutralization titers were considerably lower when interfering V3 peptides were present, indicating that V3 specificities constituted a substantial proportion of the total SF162 neutralization activity in these rabbit sera. In fact, few sera neutralized SF162 when V3 peptides were present. However, the sera from rabbit D303 (Env) and E307 (ΔV1V2.2) showed some activity in the presence of V3 peptides. Only sera from rabbit G314 (ΔV1V2.9.VK) showed strong SF162 neutralization in the presence of V3 peptides, indicating that this sera contained neutralizing antibodies specific for epitopes other than the V3 loop.

### Evidence for Neo-specificities Induced by ΔV1V2.9.VK but not ΔV1V2.2

Since there were no significant differences between the immunization groups in antibody binding to gp120 or trimeric Env and neutralization of the various virus strains, we set out to investigate whether the antibody specificities induced by the ΔV1V2 mutants were qualitatively different from those induced by full-length Env. In particular, we were interested to know whether the heavily modified deletion variants induced antibodies against neo-epitopes, i.e. responses specific for the immunogens that do not react with parental Env. The induction of neo-specificities is a concern with any modified (Env) immunogen, although it is also a property that is rarely examined. One might expect that removal of the V1V2 loop creates new epitopes, for example around the V1V2 stump. Neo-epitopes may occur for some or all ΔV1V2 variants. In the one scenario, each particular variant will induce antibody specificities that preferentially recognized the homologous deletion variant in ELISA. In another scenario, the ΔV1V2 variants will induce specificities recognizing all ΔV1V2 variants more efficiently than full-length Env.

**Figure 6:**
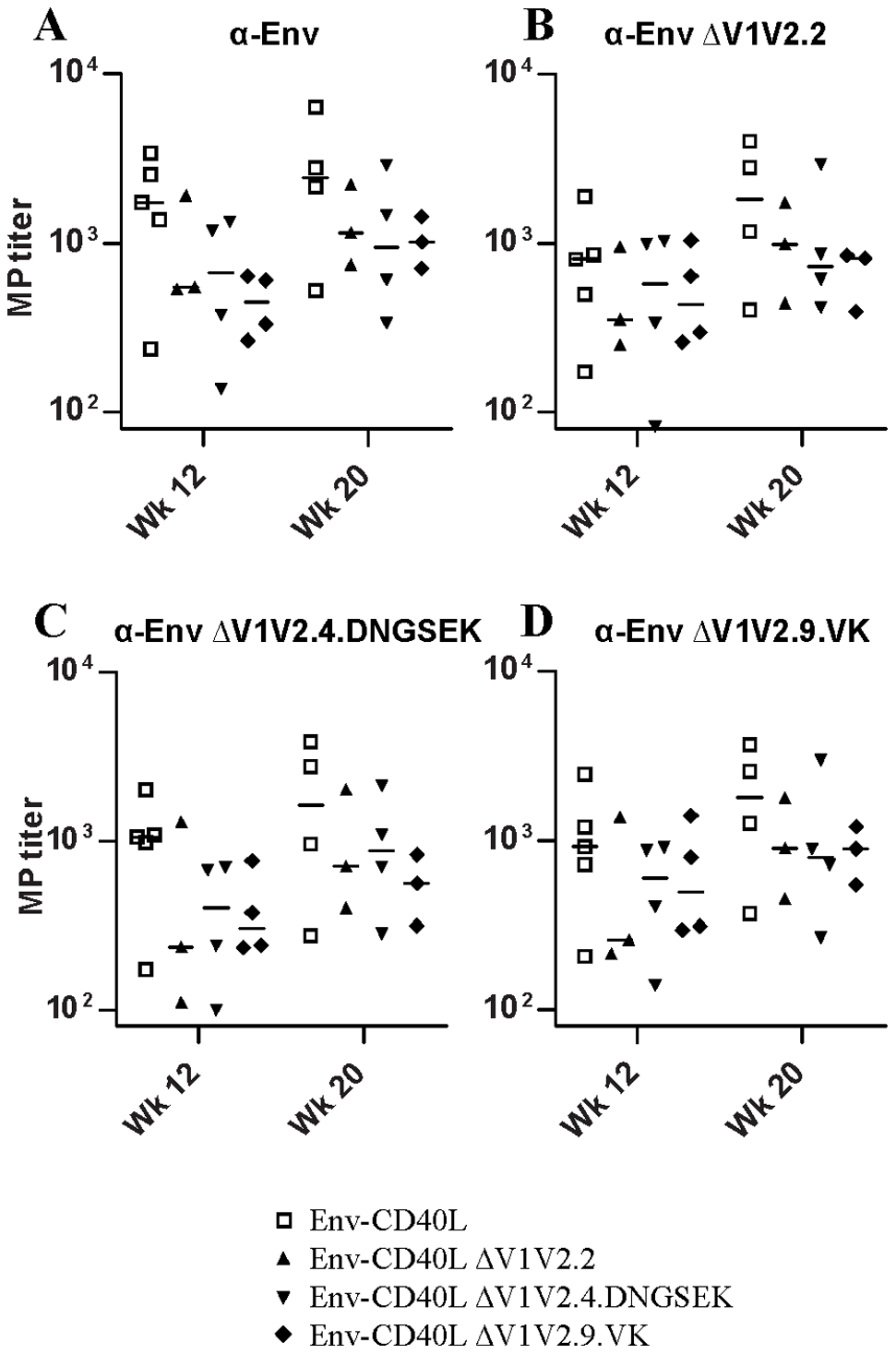
Antibody binding titers against ΔV1V2 Env. The midpoint binding titers against full length Env (**A**), ΔV1V2.2 Env (**B**), ΔV1V2.4.DNGSEK Env (**C**) or Env ΔV1V2.9.VK Env (**D**) were measured by Ni-NTA trimer ELISA.

**Figure 7:**
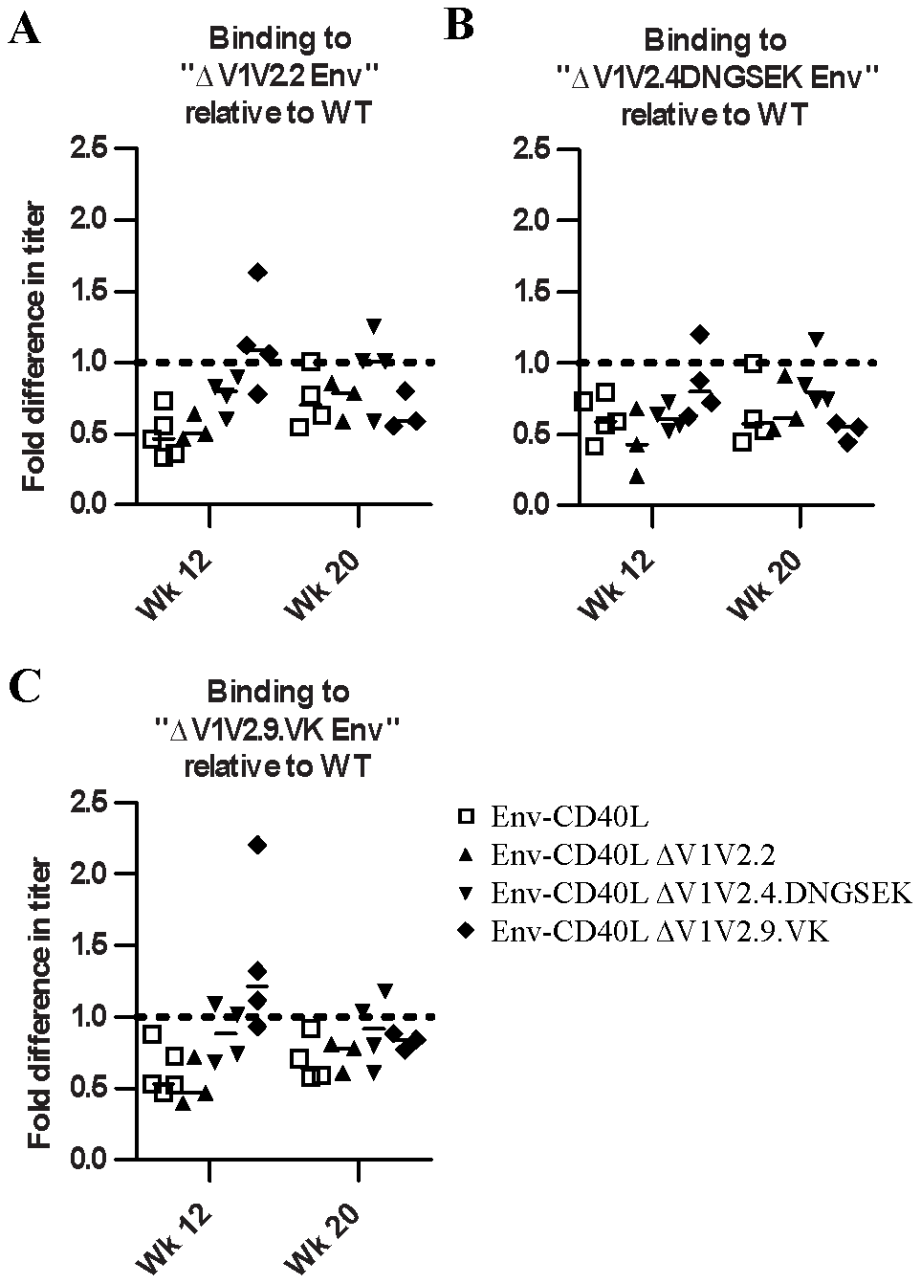
Relative antibody binding responses against ΔV1V2 Env. Each of the panels indicates the ratio of midpoint binding titers against Env ΔV1V2.2 (**A**), Env ΔV1V2.4.DNGSEK (**B**) or Env ΔV1V2.9.VK (**C**) versus the midpoint titer against full-length Env.

Above, we showed that all sera react similarly with trimeric Env in ELISA (Fig. 2D). To test whether significant neo-specificities were induced by the ΔV1V2 immunogens, we tested each serum for binding to trimeric ΔV1V2.2, ΔV1V2.4.DNGSEK and ΔV1V2.9.VK in this ELISA format (Fig. 6A–D). In general, the binding patterns were similar to the anti-gp120 and anti-trimeric Env titers determined earlier (Fig. 2). Full-length Env induced slightly higher binding titers against the ΔV1V2.2, ΔV1V2.4.DNGSEK and ΔV1V2.9.VK variants compared to the ΔV1V2 immunogens themselves, but the differences were not significant. To qualitatively assess the specificities against ΔV1V2 versus full-length Env trimers, the ratio of the midpoint binding titers to ΔV1V2 Env and full-length Env was calculated for each serum (Fig. 7A–C). This ratio can be influenced by several factors. Certain epitopes will be exposed more efficiently on ΔV1V2 compared full-length Env. We controlled for this possibility by including the “parent” sera generated against full-length Env. Furthermore, a lack of V1V2-directed specificities may lower binding and therefore the binding ratio. Conversely, an abundance of neo-specificities against ΔV1V2 Env could increase the ratio. For sera from animals immunized with full-length gp140, this ratio was ∼0.5–0.7 at both week 12 and week 18. Similar results were observed for the ΔV1V2.2 Env sera. In contrast, ΔV1V2.9.VK Env induced antibodies that recognized ΔV1V2 Env more efficiently, yielding ratios of ∼0.8–1.2 at week 12. This difference was statistically significant at week 12 for binding to ΔV1V2.2 Env by ΔV1V2.9.VK-induced sera versus full-length Env induced sera (p<0.05). Sera from ΔV1V2.4.DNGSEK-immunized animals also exhibited a slightly ΔV1V2-biased response (ratios of ∼0.6–0.9), although this difference was not statistically significant.

Interestingly, after boosting with full-length Env protein at week 16, the ΔV1V2/Env binding ratio in the sera from animals primed with the full-length, ΔV1V2.2 or ΔV1V2.4.DNGSEK Env were not significantly changed. In contrast, the ratio in the ΔV1V2.9.VK primed rabbits decreased to levels comparable to those in the other groups (0.5–0.9). This indicates that full-length Env is better recognized than ΔV1V2 Env compared to before the boost. Thus, in the ΔV1V2.9.VK primed rabbits, the booster immunization with full-length Env protein selectively boosted specificities or induced specificities *de novo*, other than or at the expense of the neo-specificities induced during the priming phase (Fig. 7).

### Neo-specificities Induced by ΔV1V2.9.VK Neutralize the ΔV1V2 Virus

We wondered whether the neo-specificities induced by ΔV1V2.9.VK Env might result in enhanced neutralization of a matched ΔV1V2 virus. To investigate, we tested these sera against a panel of LAI viruses with the exact ΔV1V2 deletions present in the index immunogens. These viruses are described in detail elsewhere [40]. It should be noted that these viruses are homologous to the immunogens in terms of the V1V2 deletions, but heterologous in terms of the Env backbone. The immunogens are based on the CCR5-using JR-FL isolate and the viruses are based on the CXCR4-using LAI isolate. The advantage of this mismatch is that we can exclude type-specific neutralizing responses against, for example, the V3 domain.

**Figure 8:**
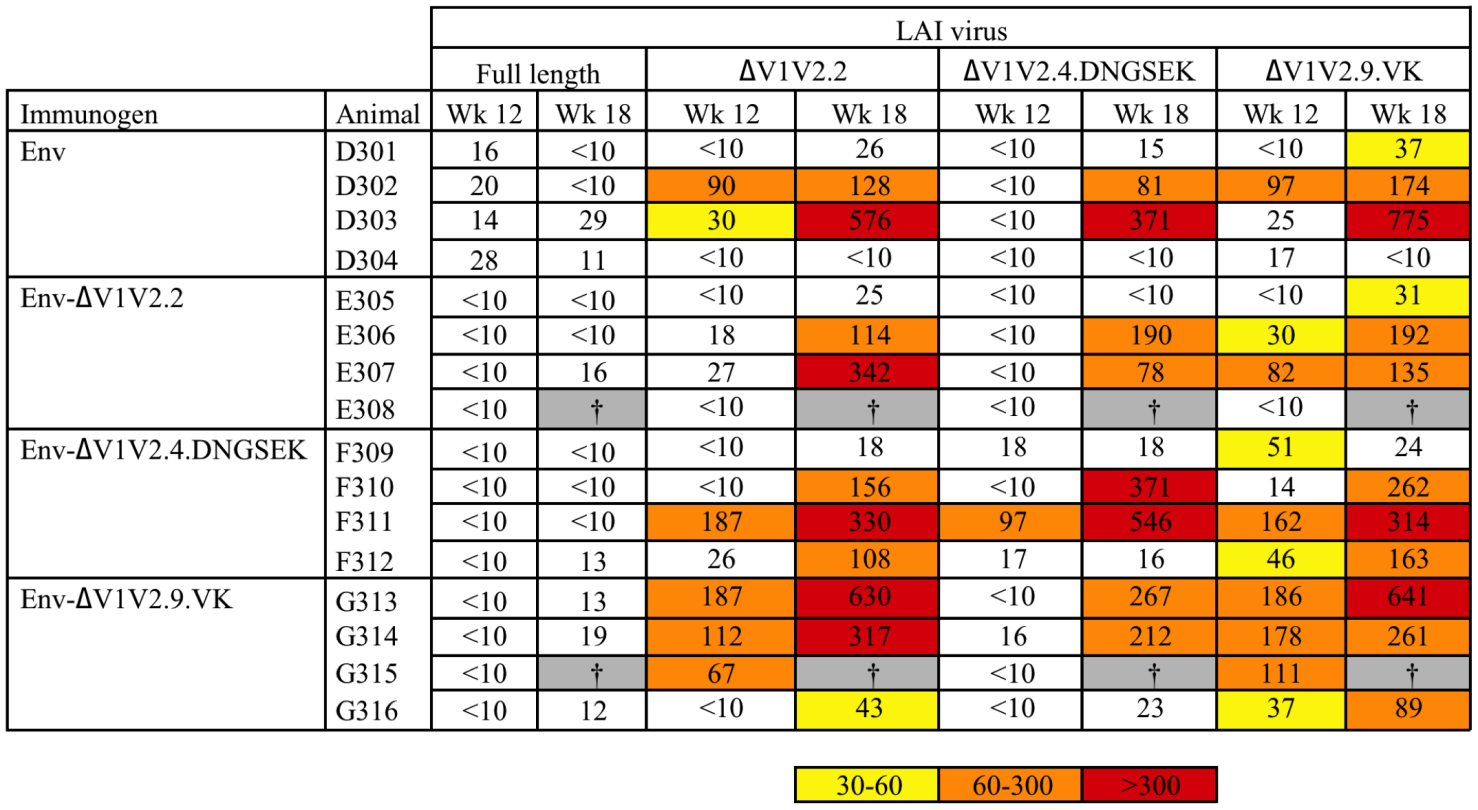
50% neutralization titers against ΔV1V2 viruses. Midpoint neutralizing titers against full length LAI, LAI ΔV1V2.2, LAI ΔV1V2.4.DNGSEK and LAI ΔV1V2.9.VK virus at week 12 and 18. Experimental conditions are similar to those in Fig. 3. The titer data are colored according to the following color scale: yellow, 50% neutralization titers between 30 and 60; orange, between 60 and 300; red, >300. The data for full length LAI data are the same as in Fig. 3. † Animals died of unrelated causes between week 12 and week 18.

We previously showed that V1V2 deletion renders the LAI virus dramatically more sensitive to neutralization by monoclonal Abs [40]. We also observed a dramatically enhanced sensitivity of the ΔV1V2 virus compared to the parental LAI strain to neutralization by the rabbit sera (Fig. 8). Consistent with the binding data (Figs. 6&7) and the presence of ΔV1V2-directed neo-specificities, the ΔV1V2.9.VK sera from week 12 (before the full-length protein boost) were most efficient at neutralizing the ΔV1V2 viruses. At week 12, 3 out of 4 sera of the ΔV1V2.9.VK group neutralized ΔV1V2.2 and ΔV1V2.9.VK virus at titers >50. In comparison, only 1/4 sera from the full-length Env and ΔV1V2.4.DNGSEK Env immunized groups and 0/4 of the ΔV1V2.2 Env immunized group were able to neutralize these viruses. None of the week 12 sera neutralized ΔV1V2.4.DNGSEK virus efficiently, except for sera from rabbit F311, which was immunized with the “homologous” ΔV1V2.4.DNGSEK Env protein. This difference was statistically significant (p<0.05). At week 18 after the protein boost, most sera neutralized the ΔV1V2.2 and ΔV1V2.9.VK viruses efficiently and about half of the sera neutralized the ΔV1V2.4.DNGSEK virus at titers >50, but no significant differences were observed between the groups primed with full-length Env or any of the ΔV1V2 variants, consistent with the binding data (Figs. 6&7). This confirms a refocusing of the antibody response induced by ΔV1V2.9.VK Env, by protein boosting with full length Env.

### ΔV1V2 Env Induces Native Trimer-binding Responses Inconsistently

**Figure 9:**
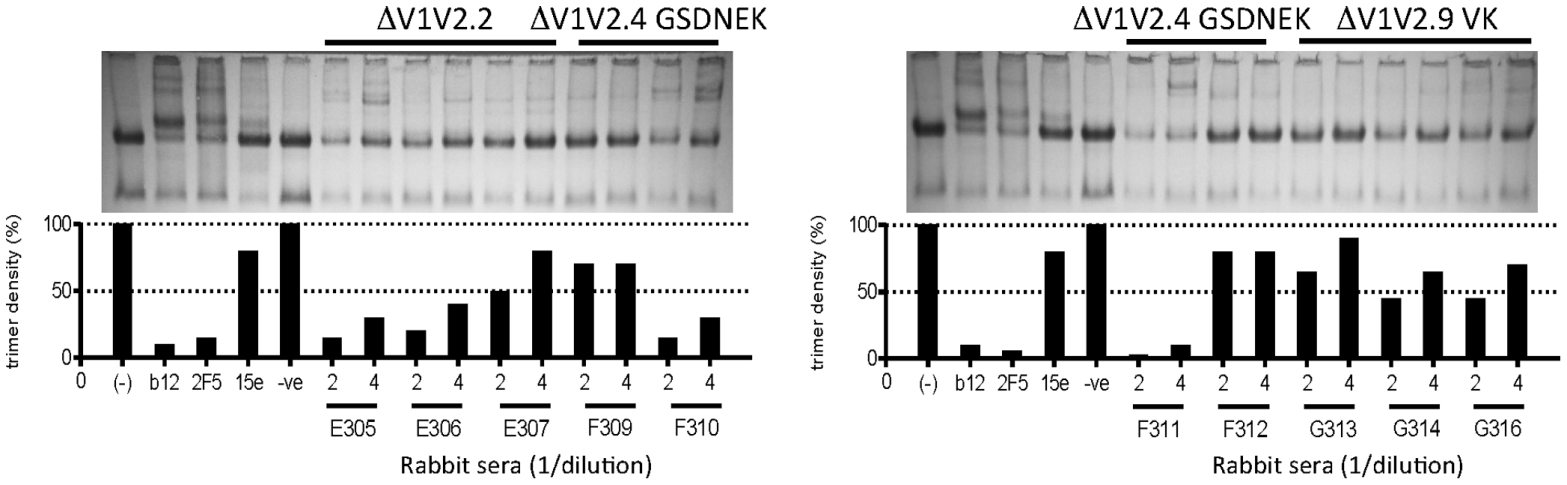
Recognition of native trimers. The binding of sera from ΔV1V2-immunized rabbits to native JR-FL Env trimers was examined. nAbs b12 and 2F5 and nonneutralizing antibody 15e served as controls. A quantitative evaluation of the trimer band intensity is presented in the bar graphs, where small bars represent efficient trimer binding of the sera and a resulting decrease in the intensity of the trimer band.

Sera from Env trimer-immunized rabbits recognize the native trimer on virus particles [50]. We investigated whether sera from ΔV1V2 Env-immunized animals could also recognize native trimers on virus particles in BN-PAGE trimer shift assays (Fig. 9) [15]. In this assay, bNAbs b12 and 2F5 efficiently depleted trimers, but the non-neutralizing antibody 15e did not (Fig. 9). The intensities of the trimer bands are represented by histograms beneath each blot: short bars indicate the presence of abundant trimer-binding antibodies that deplete the trimers, while tall bars indicate that trimer-binding antibodies were absent. Previously, we found that most sera from animals immunized with full-length Env trimers bound well to native JR-FL trimers, while sera from gp120 recipients recognized the same trimers only poorly [50]. Some of the sera from animals immunized with ΔV1V2.2 and ΔV1V2.4.GSDNEK depleted native trimers efficiently (sera E305, E306, F310, F311) while others did not (E307, F309, F312). All sera from ΔV1V2.9.VK immunized rabbits showed weak trimer depletion (G313, G314, G315). Thus, ΔV1V2 Env induces native trimer binding responses inconsistently.

## Discussion

In this study we investigated the immunogenicity of three ΔV1V2 deleted variants of the HIV-1 Env protein. These modified Env proteins were based on previous evolution, functional and biochemistry studies in which they performed optimally in terms of protein folding, expression level and Env function compared to other mutants. We studied the immunogenicity of the three selected ΔV1V2 variants and full-length Env in rabbits that were primed by DNA gene gun immunization and boosted with stabilized gp140 trimers containing the complete V1V2 domain. The rationale for investigating the immunogenicity of ΔV1V2 mutants was to improve the exposure of conserved neutralization epitopes that are (partially) shielded by the V1V2 loops [36], [38], [57], [58], [59], [60]. Thus, we hypothesized that ΔV1V2 Env variants might induce a more broadly neutralizing response compared to full-length Env. We note, however, that this hypothesis was formulated before the discovery of broadly neutralizing antibodies against the V1V2 domain [18], [19], [20], [61], and before anti-V2 antibodies were shown to correlate with vaccine protection in the RV144 trial [21], [22]. The ΔV1V2.9.VK Env variant induced antibody responses that enhanced the neutralization of ΔV1V2 viruses and the neutralization sensitive tier 1 virus SF162, but the effect was negated after boosting with full- length protein, and did not translate to more neutralization-resistant tier 2 viruses. No significant neutralization of tier 2 viruses was observed for any of the ΔV1V2-induced sera.

We found that antibody titers induced by ΔV1V2 Env against gp120 and trimeric full-length gp140 were slightly lower compared to the titers induced by full-length Env, although the results were not statistically significant (Fig. 2). These results may relate to the absence of anti-V1V2 responses. Alternatively, the slightly reduced expression level of the V1V2 variants may be the cause (Fig. 1D). It is possible that a significant fraction of antibodies is directed against neo-epitopes on and/or around the V1V2 stump that are not present or exposed on full length Env.

To explore this further for each serum we determined the ratio of antibody titers against each ΔV1V2 Env variant versus the wild-type, full-length Env (Fig. 7). We found that full-length Env induced a significant portion of antibodies that was able to bind full-length Env, but not the ΔV1V2 Env mutants, as indicated by a low ΔV1V2/Env ratio. This may suggest that a significant portion of these antibodies target the V1V2 loops, as this is the main difference between the two immunogens. Rabbits immunized with ΔV1V2.2 Env developed antibodies that produced a similar pattern to that induced by full length Env. Why these sera would recognize full-length Env better than the exact ΔV1V2.2 Env variant that was used for immunization is not clear. Thus, there was no preferential recognition of the ΔV1V2 Env mutant used for the immunization. This suggests that few antibodies induced by the ΔV1V2 Env variants target neo-epitopes on the homologous ΔV1V2 stumps. ΔV1V2.9.VK induced the highest ΔV1V2/Env ratio’s, suggesting that ΔV1V2.9.VK did induce neospecificities, but not ones that depend on the exact sequence or structure of the ΔV1V2.9.VK V1V2 stump because the ΔV1V2.2 and ΔV1V2.4.DNGSEK variants were also more efficiently recognized.

Neutralization of LAI-based ΔV1V2 virus strains was consistent with the ΔV1V2/Env antibody binding ratio’s. ΔV1V2.9.VK Env immune sera with a high ΔV1V2/Env ratio also induced a more efficient neutralization of ΔV1V2.2 and ΔV1V2.9.VK viruses at week 12. The enhanced neutralization of ΔV1V2 LAI translated to enhanced neutralization of SF162, indicating that responses are induced to regions normally shielded by the V1V2 domain, except on extremely neutralization sensitive viruses such as SF162. Consistent with the lack of ΔV1V2-specific responses induced by the ΔV1V2.2 and ΔV1V2.4.DNGSEK immunogens, the ΔV1V2/Env ratio’s did not change once the rabbits were boosted with full length Env at week 16. In contrast, we observed a decrease in the ΔV1V2/Env ratio’s for the ΔV1V2.9.VK sera upon boosting with full length Env, indicating that the ΔV1V2-focused response was lost. As a result, the improved neutralization of ΔV1V2 viruses and SF162 compared to the other immunization groups was also lost. Our rationale for boosting with a full length Env protein was to boost ΔV1V2 protein induced responses that would nevertheless recognize full length Env. Knowing the outcome of the experiment, we might have chosen a ΔV1V2.9.VK Env as the boosting protein, although it is doubtful that this would have resulted in neutralization of tier 2 viruses.

We can only speculate as to why ΔV1V2.9.VK Env induced a different response than the other two ΔV1V2 Env mutants. The SF162 virus and ΔV1V2.2 and ΔV1V2.9.VK LAI variants are more efficiently neutralized by ΔV1V2.9.VK induced sera. This suggests that the response is directed at (a) region(s) that are exposed on neutralization sensitive viruses, but not on neutralization resistant viruses. A previous study from our group indicated that ΔV1V2.9.VK is slightly more sensitive to antibodies that target the CD4 binding site (CD4BS). It could be that the CD4BS is better exposed on ΔV1V2.9.VK, leading to increased induction of CD4BS-targeting antibodies. Alternatively, the V3 may be targeted more efficiently. It is known that SF162 is more sensitive to V3 neutralization than tier 2 viruses such as JR-FL and V1V2 deletion can be accompanied by enhanced exposure of the V3 [57], [60], [62], although some studies have shown the opposite [37]. Another possibility is that the induced antibodies do not target neo-epitopes, but cryptic non-neutralizing epitopes. Thus, ΔV1V2.9.VK may redirect the responses to underlying cryptic epitopes that are available on neutralization sensitive viruses, but not on tier 2 viruses.

During the execution of these studies it was shown that the V1V2 domain harbors epitopes for broadly neutralizing antibodies such as PG9, PG16 and PGT145 [18], [19], [20], [61]. These findings may explain in part why the ΔV1V2 immunogens did not induce broadly neutralizing antibodies. Furthermore, the V1V2 domains are now known to mediate inter-protomer contacts at the apex of the Env trimer [18], [63], such that their deletion might adversely affect the overall quaternary structure of the trimer. In any case, we acknowledge that the trimers used for the priming phase in this experiment did not have an optimal structure, in that they are mostly uncleaved. We fused the trimers, via the C-terminus of gp41, to CD40L in an attempt to enhance targeting to dendritic cells and B cells [50], [51]. However, we extensions to the C-terminus of JRFL gp140 impair cleavage [49], [51]. It is now becoming clear that uncleaved gp140 trimers do not mimic the native spike in terms of both structure and antigenicity (Sanders et al. unpublished results, [33], [64], [65]).

All the above factors may have contributed to the failure of the immunogens described here to induce broadly neutralizing antibodies. We recently generated a third generation cleaved SOSIP trimer (BG505 SOSIP.664 gp140) that has improved biophysical and antigenic properties [18] Sanders et al. unpublished results). Of note is that the BG505 SOSIP.664 trimers bind very efficiently to quaternary structure dependent, broadly neutralizing antibodies against the V1V2 domain (PG9, PG16 and PGT145). It may be ill-advised to remove the V1V2 structure from these trimers because of possible adverse effects on the quaternary structure at the apex of the trimer as well as the loss of the broadly neutralizing epitopes located in this region of Env. We also note that we have recently devised ways to fuse heterologous molecules, such as CD40L, to the C-terminus of trimers without impairing their cleavage, and hence without compromising their mimicry of native Env spikes. As a result, the design of trimer-based immunogens that are directly linked to immunostimulatory molecules can now be improved beyond what we have described here.

## Materials and Methods

### Ethics Statement

Immunizations were carried out under contract by Genovac (Freiburg, Germany) at the facilities of Harlan Winkelmann (Eystrup, Germany). All animals were kept according to DIN EN ISO 9001∶2008 standards, the regulations of the German Welfare Act of 19 May 2006 (BGBI I S. 1206), the regulations of the European Union guidelines (86/609/EWG of 24 November 2006), and the European Agreement of 18 March 1986 for the protection of animal trials and other scientific studies using vertebrates (Act of 11 December 1990 [BGBI II S. 1486]). All protocols dealing with animal manipulations were in accordance with guidelines published by FELASA (Federation of European Animal Science Association) and GV-SOLAS (German Society of Laboratory Animal Science) and were reviewed by the Harlan animal care committee. The study was approved by the Landesuntersuchungsamt (Kreis Nienburg/Weser, Germany) (permit 39/30-11-1998).

### Plasmids

We have previously described modifications that improve the stability of soluble, cleaved gp140 trimers based on the R5 subtype B isolate JR-FL [33]. The amino-acid sequence of gp120 and the gp41 ectodomain was modified as follows (Fig. 1). We introduced: (i) a disulfide bond between residues 501 in gp120 and 605 in gp41 (A501C, T605C; [33]); (ii) a trimer-stabilizing substitution in gp41 (I559P; [34]); (iii) a sequence-enhanced site for furin cleavage (RRRRRR; [35]). We further modified the JR-FL SOSIP.R6 gp140 construct to include a C-terminal GCN4-based trimerization domain (isoleucine zipper; IZ) [49], [51]. We have shown that this domain further improves trimer stability. A C-terminal octa-Histidine tag (HHHHHHHHH; H8) was also added. The ΔV1V2 mutants of Env-CD40L were created by taking the previously created JR-FL SOSIP.R6-IZ-His construct with the desired ΔV1V2 mutations [40], [41] and inserting the codon-optimized active domain of rabbit CD40L downstream of isoleucine zipper (IZ) using the restriction sites for Asp718I and SfuI (Fig. 1) [50]. To enable the fair comparison of gp120 and trimeric Env, both proteins need to be capture the same way. To that end we replaced the C-terminal His tag of SOSIP.R6-IZ-His by the amino acid sequence APTKAKRRVVQREKR, the epitope for the capture antibody D7324, creating Env-D7324, as described previously [49], [50], [51].

### Cell culture and Transient Transfection

HEK 293T cells were maintained in Dulbecco’s modified Eagle’s medium (DMEM), supplemented with 10% fetal calf serum (FCS), penicillin (100 U/ml), and streptomycin (100 µg/ml) as previously described [50]. HEK 293T cells were transfected using polyethyleneimine (PEI), as described elsewhere [50]. Briefly, DNA encoding Env protein was diluted in DMEM (Invitrogen, Breda, The Netherlands), to 1/10 of the final culture volume and mixed with PEI (0.12 mg/ml final concentration). After incubation for 20 min, the DNA–PEI mix was added to the cells for 4 h before replacement with normal culture medium containing 10% FCS (HyClone, Perbio, Etten-Leur, The Netherlands) MEM nonessential amino acids (0.1 mM, Invitrogen). Culture supernatants were harvested 48 h after transfection.

### SDS-PAGE, Blue Native PAGE and Western Blotting

SDS-PAGE, blue native (BN)-PAGE, and Western blot analysis as described before [34], [50], using the JR-FL V3-specific mouse MAb PA-1 [66].

### Gene Gun DNA and Protein Immunizations

Rabbit mmunization experiments were performed together with those for another study [50], to save a control arm. Plasmid DNA was amplified using DH5α cells and isolated using the EndoFree Plasmid Giga Kit (Qiagen, Venlo, The Netherlands). The immunizations were carried out at Genovac (Freiburg, Germany), under contract. The facilities at Genovac comply with the European Community guidelines for animal housing and *in vivo* experiments (Iso 9001∶2008). Animals welfare was qualitatively assessed by an internal and external animal welfare officer (veterinarian) and by a LAVES official authority (Niedersächsisches Landesambt für Verbraucherschutz und Lebensmittelsicherheit). New Zealand white rabbits were allowed to acclimate and held in quarantine for at least 7 days prior to vaccination. The weight at the start of the experiment was ∼2.8 kg and animals were held individually in open cages (60 cm x 55 cm x 50 cm (D/L/H)) with a conventional microbiological status at a minimum temperature of 18°C. Fans were used for ventilation and the relative humidity was monitored. Day/night rhythm was not controlled and animals were exposed to natural noise and lighting. Cages were cleaned or changed twice a week and enriched with wood and hay. During the experiment animals were fed with Teklad Global Rabbit diet 2030 (Harlan Industries, Rossdorf, Germany). Water was refreshed daily and supplied *ad libitum* in bottles. Cleaning of the water bottles occurred every two weeks. Four rabbits per group were immunized and the treatment modality for each group was unknown for the staff during the entire study. On weeks 0, 2, 4 and 8 rabbits were immunized with 125 µg of endotoxin-free DNA at the abdominal dermis by ballistic gene gun technology. On week 16, all rabbits were injected with 1 ml PBS containing 30 µg purified cleaved JR-FL SOSIP.R6 gp140 protein without IZ [34], [54] and 60 µg Quil A. The injections were performed as follows: 300 µl intradermally (50 µl in each of 6 sites), 400 µl intramuscularly (200 µl into each hind leg) and 300 µl subcutaneously (neck region). Blood samples were taken from the artery down the centre of the ear, using sterile needles and tubes (Sarstedt, Nümbrecht, Germany) on weeks 0, 2, 4, 6, 8, 12, 16, 18. Time of blood sampling occurred between the hours of 8 am and 10 am. Daily checks were carried out by animal technicians and detailed health checks were carried out upon each intervention. No adverse events or reactions were noted during the experiment. On week 20, animals were sedated with a xylazine/ketamine combination (35–50 mg/kg) prior to termination. Two animals died during the experiment of unknown causes unrelated to the vaccination experiment. Note that the control arm full length Env (group D) containing the V1V2 domain, was an experimental arm (also named group D) in a study that was performed concurrently on the use of co-stimulatory molecules [50].

### Env-specific and Total Immunoglobulin ELISA

Anti-gp120 antibody titers were measured by ELISA essentially as described previously [49]. Anti trimeric gp140 titers were measured using the Env-D7324 construct [49], [50]. For measuring total serum immunoglobulin levels goat anti-mouse IgG (Jackson ImmunoResearch, Newmarket, UK) was coated overnight (10 µg/ml) in 0.1 M NaHCO_3_, pH 8.6 (100 µl/well). After blocking, serially diluted serum was applied for approximately 2 h. Bound rabbit IgG was detected with HRP-labeled goat anti-Rabbit IgG (Jackson Immunoresearch, Suffolk, England; used at 1∶5000 (0.2 µg/ml)), followed by luminometric detection. Midpoint titers were calculated using Graphpad Prism version 5.03 by determining the dilution of the serum at which the optical density was 50% of maximum.

### ΔV1V2 Env ELISA

The C-terminal His tag on SOSIP.R6-IZ-His (Fig. 1) and the ΔV1V2 mutants was used to specifically capture these molecules from transiently transfected cell supernatant onto Ni-NTA coated Hissorb 96-well plates (Qiagen, Venlo, The Netherlands). After 2 hour capture, the wells were washed 3× using TSM (20 mM Tris, 150 mM NaCl, 1 mM CaCl_2_, 2 mM MgCl_2_), followed by a 2 hr incubation with the rabbit sera, serially diluted in SS/milk (TBS/20% sheep serum/1.6% milk). After washing 5× with TSM 0.05% Tween 20, the wells were incubated for 1 hour with 1∶5000 diluted HRP-labeled Goat-anti-Rabbit IgG (Jackson) in TSM 5% BSA. The wells were then washed with TSM/0.05% Tween 20 and developed and stopped as described above.

### Neutralization Assays

The TZM-bl reporter cell line stably expresses high levels of CD4 and HIV-1 co-receptors CCR5 and CXCR4 and contains the luciferase and β-galactosidase genes under the control of the HIV-1 long-terminal-repeat promoter. The TZM-bl cell line was obtained through the NIH AIDS Research and Reference Reagent Program, Division of AIDS, NIAID, National Institutes of Health (John C. Kappes, Xiaoyun Wu, and Tranzyme Inc. (Durham, NC)). Single-cycle infection experiments and inhibition experiments using TZM-bl cells were performed as described [49]. Midpoint neutralizing titers of the sera were determined by determining at which serum dilution there was 50% of the luciferase activity compared to no serum. The percentage of neutralization was determined by measuring how much of the luciferase signal was lost compared to no serum. The viruses tested at the Duke Central Immunology Laboratory for AIDS Vaccine Research and Development were the tier 1 strains MN, SF162.LS and BaL.26 and the tier 2 strains JR-FL, 6535.3, QH0692.42, PVO.4 and RHPA4259.7. The sera were heat inactivated (30 min 56°C) before use.

### IgG Depletion

120 µl of serum was mixed with 200 µl 50% slurry of Pierce protein G plus agarose (Pierce/Thermo Fischer, Etten-Leur, The Netherlands) and 780 µl phosphate buffered saline pH 7.4. This was mixed overnight at 4°C after which thorough washing using 1X RIPA buffer. Rabbit IgG was eluted from the beads using 550 µl IgG elution buffer (Pierce) and immediate neutralization with 50 µl 1 M Tris-HCl pH 9.5.

### V3 Depletion

V3 peptide competition experiments. Rabbit sera were serially diluted and preincubated with a mix of three overlapping peptides (20 µg/ml of each) spanning the JR-FL V3 region (Env V3-1 [NNNTRKSIHIGPGRA], Env V3-2 [SIHIGPGRAFYTTGE], and Env V3-3 [GRAFYTTGEIIGDIR]) or with an unrelated peptide (QAPKPRKQ [60 µg/ml]) for 1 h at room temperature. The peptide-serum mixtures were then tested for HIV-1 neutralization. To control for non-specific inhibition by the peptides, we also performed neutralization assays using MAb b12 (6.0 µg g/ml) in the presence or absence of the three V3 peptides; they did not inhibit b12 neutralization (data not shown).

### Trimer Shift Assays

BN-PAGE trimer shift assays. Blue native PAGE (BN-PAGE) analyses were performed as described previously [14], [15], [67]. Briefly, virus-like particles (VLPs) bearing wild-type (wt) JR-FL trimers were incubated with MAbs or sera, washed, and gently solubilized in 0.12% Triton X-100, 1 mM EDTA, 1.5 M aminocaproic acid with a protease inhibitor cocktail containing 4-(2-aminoethyl)benzenesulfonyl fluoride, E-64, bestatin, leupeptin, aprotinin, and sodium EDTA (P-2714; Sigma). An equal volume of sample buffer (100 mM morpholinepropanesulfonic acid [MOPS], 100 mM Tris-HCl [pH 7.7], 40% glycerol, 0.1% Coomassie blue) was added. Samples were loaded onto a 4% to 12% Bis-Tris NuPAGE gel (Invitrogen) and separated at 4°C for 3 h at 100 V. Ferritin (Amersham) was used as a size standard. The gel was then blotted onto a polyvinylidene difluoride membrane that was destained, immersed in blocking buffer (4% nonfat milk–PBS), and probed with a cocktail consisting of MAbs 2G12, b12, E51, 39F, 2F5, 4E10, 7B2, and 2.2B followed by an anti-human Fc alkaline phosphatase conjugate (Jackson) and SigmaFast BCIP/NBT (5-bromo-4-chloro-3-indolyl phosphate/nitroblue tetrazolium) substrate (Sigma).

### Statistical Analyses

All statistical analyses were performed using GraphPad Prism 5.03. One-tailed Mann-Whitney U tests were used to analyze immunogenicity data. Kruskal-Wallis tests were also performed to compare the same groups, followed by Dunn’s multiple comparison test in cases in which the medians were found to be significantly different, unless indicated otherwise.
